# Phylogeny and Optimization of *Trichoderma* *harzianum* for Chitinase Production: Evaluation of Their Antifungal Behaviour against the Prominent Soil Borne Phyto-Pathogens of Temperate India

**DOI:** 10.3390/microorganisms9091962

**Published:** 2021-09-15

**Authors:** Fayaz A. Mohiddin, Shahid A. Padder, Arif H. Bhat, Mohammad A. Ahanger, Asif B. Shikari, Shabir H. Wani, Farooq A. Bhat, Sajad Un Nabi, Aflaq Hamid, Nazir A. Bhat, Najeebul R. Sofi, Showkat A. Waza, Burhan Hamid, Shugufta Parveen, Ashaq Hussain, Aadil N. Bhat, Omar M. Ali, Mohammad S. Dar, Arafat Abdel Hamed Abdel Latef

**Affiliations:** 1 Mountain Research Centre for Field Crops, Sher-e-Kashmir University of Agricultural Sciences and Technology of Kashmir, Srinagar 192124, India; asifshikari@gmail.com (A.B.S.); nazirpathology@gmail.com (N.A.B.); najeeb_sofi@rediffmail.com (N.R.S.); ahshah71@gmail.com (A.H.); aadilnazir624@gmail.com (A.N.B.); saleemdar0001@gmail.com (M.S.D.); 2 Division of Basic Sciences & Humanity, Faculty of Horticulture, Sher-e-Kashmir University of Agricultural Sciences and Technology of Kashmir, Srinagar 192125, India; 3 Mountain Crop Research Station, Sagam, Sher-e-Kashmir University of Agricultural Sciences and Technology of Kashmir, Srinagar 192124, India; mashrafjs@gmail.com (M.A.A.); sahmad777@gmail.com (S.A.W.); 4 Division of Plant Pathology, Faculty of Agriculture, Wadura, Sopore, SKUAST-Kashmir, Sopore 193201, India; farooqahmad_bhat@ymail.com (F.A.B.); shugufta1351@gmail.com (S.P.); 5 Plant Pathology, Central Institute of Temperate Horticulture, Srinagar 190007, India; sajadnabi21@gmail.com; 6 Division of Plant Pathology, Faculty of Horticulture, Sher-e-Kashmir University of Agricultural Sciences and Technology of Kashmir, Srinagar 192125, India; falak19@gmail.com; 7 Centre of Research for Development, University of Kashmir, Srinagar 190006, India; peerzada19@gmail.com; 8 Department of Chemistry, Turabah University College, Turabah Branch, Taif University, Taif 21944, Saudi Arabia; om.ali@tu.edu.sa; 9 Botany and Microbiology Department, Faculty of Science, South Valley University, Qena 83523, Egypt; moawad76@gmail.com

**Keywords:** *Trichoderma*, characterization, phylogeny, chitinase, soil borne, phytopathogens, bioagents

## Abstract

*Trichoderma* is the most commonly used fungal biocontrol agent throughout the world. In the present study, various *Trichoderma* isolates were isolated from different vegetable fields. In the isolated microflora, the colony edges varied from wavy to smooth. The mycelial forms were predominantly floccose with hyaline color and conidiophores among all the strains were highly branched. Based on morphological attributes, all the isolates were identified as *Trichoderma harzianum.* The molecular identification using multilocus sequencing ITS, *rpb2* and *tef*1α, genes further confirmed the morphological identification. The average chitinase activity varied from 1.13 units/mL to 3.38 units/mL among the various isolates, which increased linearly with temperature from 15 to 30 °C. There was an amplified production in the chitinase production in the presence of Mg^+^ and Ca^2+^ and Na^+^ metal ions, but the presence of certain ions was found to cause the down-regulated chitinase activity, i.e., Zn^2+^, Hg^2+^, Fe^2+^, Ag^+^ and K^+^. All the chitinase producing *Trichoderma* isolates inhibited the growth of tested pathogens viz., *Dematophora necatrix, Fusarium solani, Fusarium oxysporum* and *Pythium aphanidermatum* at 25% culture-free filtrate concentration under in vitro conditions. Also, under in vivo conditions, the lowest wilt incidence and highest disease control on *Fusarium oxysporum* was observed in isolate BT4 with mean wilt incidence and disease control of 21% and 48%, respectively. The *Trichoderma harzianum* identified in this study will be further used in formulation development for the management of diseases under field conditions.

## 1. Introduction

The genus *Trichoderma* is the most important filamentous fungi in biological control strategies of phytopathogen management [1,2]. Soil microflora, especially the genus *Trichoderma,* has predominantly been isolated from the rhizospheric soils [3]. *Trichoderma* species plays an important role in inhibiting the activity of up to 80% of some economically important plant pathogens [4] and nematodes [2] and because of their diversity in the production of a plethora of metabolites, they are considered to be potential candidates in the biocontrol apparatus of plant disease management [5].

The genus *Trichoderma* brings about the phytopathogen degradation by innumerable means, and chitinase activity is one of the imperative aspects in the process of its antifungal behavior [6] besides jasmonic acid-mediated systemic resistance [7]. Chitin, an insoluble linear β-1, 4 linked homopolymer of N-acetylglucosamine, is one of the most abundant natural renewable compounds. It is a polysaccharide chemically associated with cellulose [8]. It is a prominently abundant polysaccharide in major fungi cell walls viz., ascomycetes, chitridiomycetes, basidiomycetes and deuteromycetes, insect exoskeleton and crustacean shells [9]. Various types of microorganisms, viz., fungi, bacteria, yeast, etc., produce chitinase enzymes for catalyzing the biological hydrolysis of chitin to its monomer N-acetyl-D-glucosamine [10,11]. The chitinase enzyme produced by different biological entities digest chitin and utilizes it as carbon and energy sources [12]. Due to the chitin-based cell wall degrading nature of the chitinase enzyme, it is most promptly used in biological research as a controlling agent for the generation of fungal protoplast [13]. Chitinase mediated processing of shellfish helps to minimize the chitin waste and improve the product’s biological properties [14]. Chitinase enzymes can be pivotal in plasticizing the host cell wall during its biological parasitism of phytopathogens or can act more specifically during cell separation, nutritional chitin acquisition, or competitive interaction with other fungi [15]. Each year, a large quantity of chitin waste material is released into the environment, creating a serious environmental concern [16]. 

The recycling of chitin is needed for retaining the carbon-nitrogen balance in the ecosystem [17]. Medium optimization for chitinase production not only is less time consuming but capable of detecting the true optimum level of the factor involved in its production process [18]. In the farming industry, the *Trichoderma* sp., besides phytopathogen management, has directly or indirectly been used as a central biological tool for insect pest management practices [19] and stress mitigating entities [20]. Therefore, the aim of the present study was not only to isolate and characterize various *Trichoderma* strains but to optimize the production of chitinase by evaluating various physical and nutritive parameters.

## 2. Materials and Methods

### 2.1. Isolation and Identification of Trichoderma *spp.*

Rhizospheric soil samples were collected from various vegetable kitchen gardens and commercial chilli fields (20 each) from different geographical regions of the Kashmir valley (J&K, India) viz., Anantnag (3980 Km^2^ area, 34.830N 75.250E, 5300 feet elevation) and Baramulla (4599 Km^2^ area, 33.20N 73.340E, 5220 feet elevation). The samples were brought to the laboratory in sterile polypropylene bags. The soil suspensions (0.1 mL) of the 10^−4^ dilution were inoculated in Petri dishes containing *Trichoderma* specific media (TSM) (Composition gL-MgSO^4^.7 H2 O, 0.2; K_2_HPO^4^, 0.9; KCl, 0.15; NH_4_NO_3_, 1.0; glucose, 3.0; chloramphenicol (Chloromycetin, Sigma Aldrich Co., 3050 Spruce St. 63103 St. Louis USA), 0.25; p-dimethylamino benzene diazo sodium sulfonate (Dexon 60% w.p., Farbenfabrik Bayer A.G., Wuppertal, Germany), 0.3; pentachloronitrobenzene (Terraclor 75 w.p., Olin Chemicals, Houston, TX, USA), 0.2; rose-bengal (tetra chloro tetra diodofluorescein, BDH Chemicals Ltd., England), 0.15; agar (Difco Laboratories, Franklin Lakes, NJ, USA). The plates were incubated at 28 °C, and the resultant colonies were allowed to grow up to 48 h. The individual colonies were transferred separately to the Petri plates containing the same medium (TSM) and incubated for up to five days until sufficient growth was achieved. A total of 110 isolates were collected (which includes 83 from kitchen gardens and 27 from commercial chilli fields). All the isolates were identified on the basis of macro morphological (colony appearance, colour and growth rate) and micromorphological (conidial shape and size; phialide shape and size) characteristics.

### 2.2. Chitinase Activity

In order to screen the isolated *Trichoderma* spp. for chitinase activity, the strains were grown in 100 mL of fresh minimal medium fortified with colloidal chitin [2.0 g colloidal chitin, 0.1 g KH_2_PO_4_; 0.01 g MgSO_4_.7H_2_O; 3.0 g NaCl; 0.7 g (NH_4_)_2_SO_4_; 0.05 g yeast extract; and 50 mM of sodium phosphate buffer, pH 6.0 (all the components are given on per liter basis] in 250 mL Erlenmeyer flasks at 30 °C over three days. After three days of incubation, supernatant (enzyme solution) was collected by centrifuging the mixture for 20 min at 12,000 rpm.

The method of [21] with some modifications was used to calculate chitinase activity. Colloidal chitin was selected as a sole substrate. The substrate and enzyme mixture [0.5 mL (1% *w/v* of colloidal chitin) and 0.5 mL of enzyme solution] was incubated at 45 °C for 1 h. The reaction was then stopped by 3 mL of a 5-dinitrosalicylic acid reagent and then heated for five minutes at 100 °C to completely cease the reaction. The supernatant (10,000 rpm for 15 min) was collected and subjected to absorbance measurement with spectrometer UV (530 nm). For determination of enzyme unit, serial dilutions of N-acetyl glucosamine from 0 to 50 mM) were prepared. One unit (U) of the chitinase activity was defined as the amount of enzyme required to release 1 mmol of N-acetyl D glucosamine (as a standard) from chitin/min.

### 2.3. Optimization of Chitinase Production

The optimization studies on chitinase production as influenced by various substrates, chemicals and environmental conditions were carried out (in triplicates) in the following fashion:

#### 2.3.1. Colloidal Chitin Concentration

Erlenmeyer flasks (250 mL), each containing 50 mL of minimal media amended with different substrate (colloidal chitin) concentrations viz. 0.5%, 1.0%, 1.5%, 2.0% and 2.5% were prepared. The pH of the medium was adjusted to 4.7, and all the flasks were autoclaved at 121 °C for 15 min. Each of the flasks was inoculated with 0.5 mL of spore suspension (1 × 10^6^/mL) of *Trichoderma* and incubated in a rotary shaker incubator with 140 rpm at 30 °C for 5 days. Culture filtrate was harvested after 5 days, and the enzyme activity was assessed, as discussed previously in Section 2.2.

#### 2.3.2. Effect of pH and Temperature

In order to study the effect of pH on the chitinase production process, nine Erlenmeyer flasks (250 mL) marked as A, B, C, D, E, F, G, H, I and J each containing 50 mL of minimal medium fortified with colloidal chitin were prepared (in triplicates) and pH of each flask was kept different from each other to see the effect on chitinase production. The selected pH levels studied included 3.0, 3.5, 4.0, 4.5, 5.0, 5.5, 6.0, 6.5 and 7.0 in A, B, C, D, E, F, G, H, I and J, respectively, with each flask representing a different pH level. The inoculation, incubation and other conditions remained the same as in Section 2.3.1.

In order to visualize the effect of temperature on chitinase production, the minimal media was prepared in seven Erlenmeyer flasks (250 mL), as discussed in Section 2.2. All conditions as reflected in the standard protocol for chitinase production assay were kept fixed for all the flasks except for the temperature of incubation. Each flask was kept at a unique incubation temperature and thus formed seven experimental units with temperature levels of 15, 20, 25, 30, 35, 40, 45 °C in flasks A, B, C, D, E, F and G, respectively. The steps on inoculation and calculation of chitinase activity were the same as in Section 2.2.

#### 2.3.3. Effect of Nitrogen and Carbon Sources

In order to investigate the effect of nitrogen and carbon sources on chitinase production in *Trichoderma* sp., the six experimental units in triplicates were set (Erlenmeyer flasks (250 mL) each containing 50 mL minimal media as in Section 2.2). Each flask was marked as A, B, C, D, E and F and was amended with 1% of nitrogen sources in fashion as*:* peptone in flask A, tryptone in flask B, casein in flask C, urea in D, (NH_4_)_2_SO_4_ in E and NaNO_3_ in flask F. The inoculation, incubation and other conditions remained the same as in Section 2.3.1. Culture filtrate was harvested after 5 days, followed by supernatant collection and the enzyme activity measured, as discussed previously in Section 2.2.

The effect of various carbon sources on the chitinase production process was investigated by preparing the minimal media as in Section 2.2. 50 mL minimal media was taken in Erlenmeyer flasks (250 mL) marked as A, B, C, D, E, F, G, H, I and J. Each of the flasks was amended with 1% of different carbon sources viz., glucose in flask A, sucrose in B, galactose in C, arabinose in D, raffinose in E, mannose in F, maltose in G, fructose in H, lactose in I and xylose in flask J. All other conditions for chitinase production remained the same as per the protocol 2.3.1. The calculations on per unit enzyme production were the same as in Section 2.2.

#### 2.3.4. Effect of Metal Ions

In order to evaluate the effect of metal ion amendments on chitinase production, Erlenmeyer flasks (250 mL) each containing 50 mL of minimal media as in Section 2.2. were prepared and marked as 1, 2, 3, 4, 5, 6, 7 and 8. Each flask was amended with 10 mM conc. of one of the cations in the manner as appended; K^+^ in flask 1, Ag^+^ in 2, Ca^2+^in 3, Fe^2+^in 4, Hg^2+^ in 5, Mg^2+^ in 6, Na^+^ in 7 and Zn^2+^in flask 8. Each of the isolates was separately inoculated on all the amended media and incubated as per the protocol, and the steps for calculation of chitinase activity were the same as in Section 2.2.

### 2.4. Molecular Characterization

The selected isolates representative of different geographical regions was also characterized at a molecular level through multi-gene sequencing.

#### 2.4.1. Genomic DNA Extraction

For isolation of genomic DNA, 17 randomly selected isolates of *Trichoderma* representing different geographic regions were grown on Potato Dextrose Broth (PDB) for 7 days. The harvested mycelia were dried and frozen immediately in liquid nitrogen. The DNA was extracted from 100 mg mycelium using CTAB Cetyl trimethyl ammonium bromide) method with some modifications as described by [22]. The DNA was qualitatively analyzed in 1% agarose gel.

#### 2.4.2. PCR Amplification

All 17 isolates were amplified using primers of three genes, viz., *its* (Internal transcribed spacer), *rpb2* (RNA Polymerase B gene) and *tef*1α (Translation elongation factor-1α). All 17 isolates were amplified using ITS1 as forward and ITS4 as a reverse primer using Polymerase chain reaction (PCR) in a thermal cycler. The *rpb2*gene with primer pair of *RPB2_210up* (TGCGGWGAYCARAARAAGG) and *RPB2_145low* (CATRATGACSGAATCTTCCTGGT) were used in touchdown amplification protocol as: 3 min initial denaturation at 94 °C, 5 cycles each of 45 sec at 94 °C, 45 sec at 60 °C and 2 min at 72 °C followed by 5 cycles with the annealing temperature decreasing by 0.1 °C per cycle from 58 °C to 54 °C followed by 30 cycles with annealing at 54 °C and with a final extension of 10 min at 72 °C (Liu et al. 1999). The *tef*1α (primer pair of *tef71*F (CAAAATGGGTAAGGAGGASAAGAC) and *tef997*R (CAGTACCGGCRGCRATRATSAG) were also used in touchdown amplification protocol as: 4 min initial denaturation at 94 °C, 4 cycles each of 1 min at 94 °C, 90 s at 70 °C and 90 s at 72 °C, followed by 26 cycles with the annealing temperature decreasing by 0.5 °C per cycle from 68 °C to 55 °C, followed by 12 cycles with annealing at 55 °C and with a final extension of 7 min at 72 °C [23]. The reaction was set up according to the protocol [24]. The amplicons were observed under UV in the gel-documentation system (Bio-Rad, Gel Doc XR system).

#### 2.4.3. Sequencing and Phylogenetic Analysis

The amplified PCR products were custom sequenced at Xcelris Labs (Ahmedabad Gujarat, India). The DNA baser V.4 program was used to assemble the sequences of both forward and reverse reactions to produce complete contig. The BLASTn was performed for all the contigs from NCBI (http://ncbi.nlm.nih.gov/BLAST) to search the homologous sequences. The generated sequences from the current study and reference sequences from GenBank were used to construct a phylogeny by a maximum likelihood method (MLM) with 1000 replications for each bootstrap value using the MEGA 10 software version [25].

### 2.5. Antagonistic Activity of Cell-Free Culture Filtrates against Soil Borne Pathogens

The cell-free culture filtrate was prepared from the chitinase producing strains in Potato Dextrose Broth (PDB) (Himedia Laboratories Pvt. Ltd., India) and incubated at 28 °C on a shaker incubator (150 rpm). Cell-free supernatants were collected after 10 days of incubation by aseptic filtration through Whatman filter paper No. 1 followed by re-filtration through a 0.20 µm cellulose acetate syringe filter. The growth inhibitory effects of extracellular metabolites from culture filtrates were estimated by using radial growth inhibition assay on PDA amended with 5, 10, 15, 20 and 25% (*v/v*) cell-free filtrate against *Fusarium* *oxysporum, Dematophora necatrix, Pythium amphanidermatum*. Fungal growth inhibition was expressed as a percentage of radial growth inhibition relative to the control [26]. Also, the culture filtrate effect of *Trichoderma* isolates on inhibition of *Fusarium* *oxysporum* was studied in a pot culture experiment. Pots of 15 cm diameter were surface sterilized with 1% sodium hypochlorite and filled with 500 g of an autoclaved mixture of soil and sand (5:1). A single seedling (two weeks old) of chilli plant grown in sterilized soil was transplanted in each pot. Four plates of *F.* *oxysporum* culture grown on PDA were scraped with a sterilized spatula and mixed with sterilized distilled water to obtain 1 × 10^6^ CFU/mL spore suspension. *F.* *oxysporum* spore suspension (10 mL) was added around the plant root in the soil. Spore suspension of *Trichoderma* isolates (50 mL, 1 × 10^6^ CFU/mL) was mixed with the pathogen infested soil separately. Pots inoculated with only distilled water served as control. Six replicates were prepared for each treatment. Plants were irrigated with sterile water, as per requirement and wilt incidence was recorded at different growth stages.

### 2.6. Statistical Analysis

All the experiments were conducted in a completely randomized design (CRD), and the experimental data are expressed as mean ± SD from five separate observations/replications. Normality and homogeneity of the collected data were tested using Levene’s Test in IBM SPSS Statistics 19.0 (SPSS, Inc., Chicago, IL, USA). The statistical significance of the treatments was determined by Tukey’s test in XLSTAT (v.2021.3.1).

## 3. Results

### 3.1. Screening and Morphological Identification of Trichoderma Isolates

A total of 110 *Trichoderma* isolates were collected from different geographical regions of Kashmir, India. However, the detailed studies were carried out only on selected isolates based on their ability to inhibit the growth of potential chilli pathogen (*Fusarium solani*) of the area. At 25 °C on potato dextrose agar plates, the colonies varied from light green, snow green to dark green and became compact with time. The colony edges varied from wavy to smooth, mycelial forms and color varied from floccose to arachnoid and hyaline, respectively, conidiophores were branched. Based on morphological attributes, all the isolates were identified as *Trichoderma harzianum* (Table 1).

### 3.2. Screening for Chitinase Activity

The results on screening of chitinolytic strains revealed that the chitinase activity varied between 1.13 units/mL in *Trichoderma* isolate (AT2) to 3.38 units/mL in Trichoderma isolate (BT3). All the isolates differed significantly from each other in post hoc analysis by Tukey’s test (Table 1).

### 3.3. Molecular Identification and Phylogenetic Analysis

To confirm the identity of genus and species at the molecular level, the ITS region was amplified in 17 *Trichoderma* isolates and the amplicon of 550 bp obtained after PCR amplification was sequenced. The sequence data of isolates after BLASTn analysis showed a maximum similarity of 99–99.5% with *Trichoderma harzianum* (NR131264) from type material present in NCBI GenBank except isolates AT2, BT6, BT7, BT8 and BT9. To further validate the ITS results, two genes, *rpb2* and *tef*1α, were used, which amplified the products of 1000 bp and 800 bp respectively from all the isolates. The *tef*1α gene results showed sequence similarity of 98% with *T. harzianum* and whereas *rpb2* showed 99% with *Trichoderma afroharzianum* (cryptic species of *Trichoderma harzianum),* respectively.

**Figure 1:**
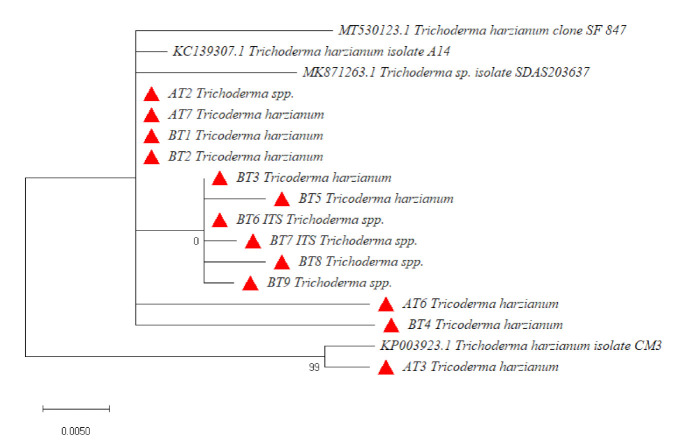
Phylogenetic relationship of *Trichoderma* spp using the internal transcribed spacer (ITS) region gene nucleotide sequence alignment using maximum likelihood method at 1000 replications for each bootstrap value using the MEGA 10.0, symbols in red triangles phylogeny represents our isolates.

**Figure 2:**
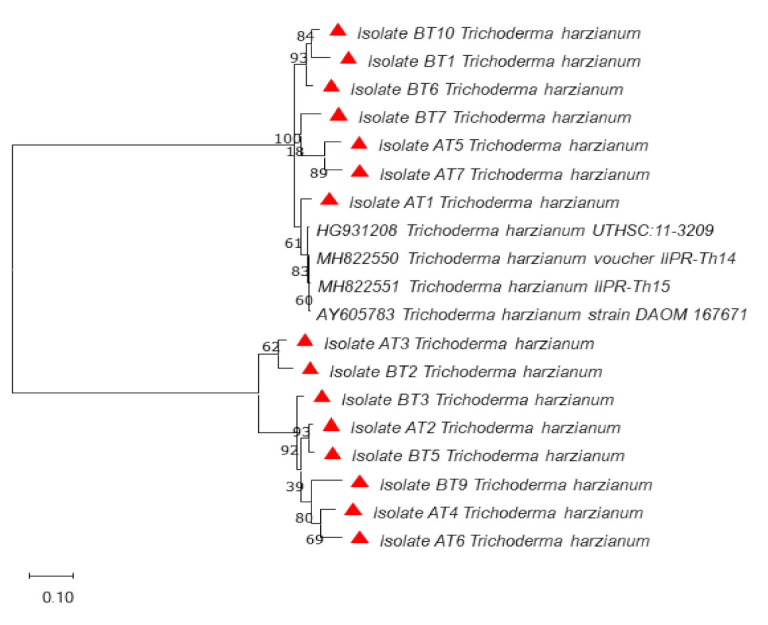
Phylogenetic relationship of *Trichoderma harzianum* using the region *tef*1α (Translation elongation factor-1α) gene nucleotide sequence alignment using maximum likelihood method at 1000 replications for each bootstrap value using the MEGA 10.0, symbols red triangles in phylogeny represents our isolates.

**Figure 3:**
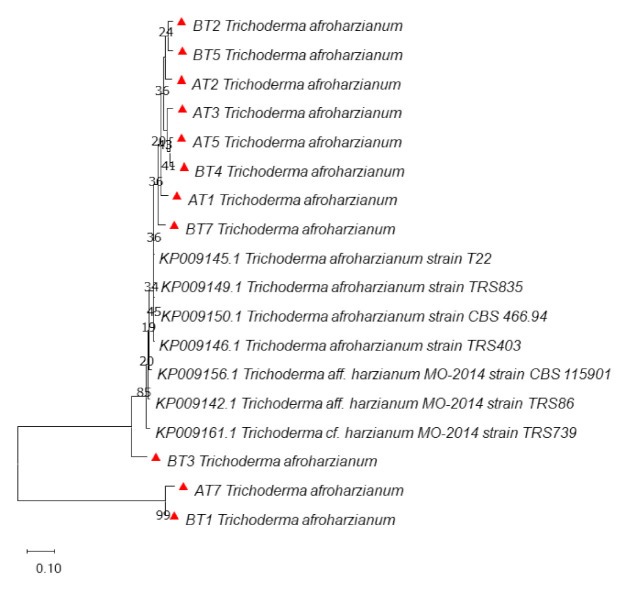
Phylogenetic relationship of *Trichoderma harzianum* using the region *rpb2* (RNA Polymerase B) gene nucleotide sequence alignment using maximum likelihood method at 1000 replications for each bootstrap value using the MEGA 10.0, symbols red triangles in phylogeny represents our isolates.

Phylogenetic studies using DNA sequence data are proving highly effective in distinguishing species. Additional sequences retrieved from the GenBank were used in the analysis of DNA sequences data of *Trichoderma* isolates. The phylogenetic tree from ITS data did not provide good resolution among *Trichoderma* isolates. It provided resolution only up to the genus level in some of the isolates (AT2, BT6, BT7, BT8 and BT9). The phylogenetic analysis of the ITS gene showed that all the isolates of *Trichoderma* clustered in one group except BT4 and BT5, which clustered separately (Figure 1) shows the presence of diversity at the genus level. The reference sequences clustered separately with only AT3 isolate clustering with them, indicating that the characterized isolates are not exactly matching with the reference sequences. The analysis with *tef*1α divided the isolates into two main clusters. All the reference isolates separated into one cluster, while as all the *Trichoderma* isolates formed one cluster. The *Trichoderma* isolates were further divided into two sub-clusters, except BT7, which separated from both the sub-clusters. All the isolates in both the clusters were found identical to *Trichoderma harzianum* (Figure 2). Hence *tef*1 was successful in providing the resolution up to species level. *In rpb2* phylogenetic analysis, AT7, BT1 and BT3 clustered with reference sequences while all other isolates formed a single cluster. From this cluster, BT7 separated as an independent lineage (Figure 3). However, *rpb2* sequence analysis showed that all the isolates were identical to *Trichoderma afroharzianum,* which is a cryptic species of the group *T. harzianum*. The multi-gene approach confirmed a significant increase in the power of discrimination and the robustness of the phylogenetic tree, which made it possible to provide an accurate molecular basis for species or cryptic species identification among the *Trichoderma* isolates.

### 3.4. Optimization of Chitinase Producing Trichoderma Isolates

All the isolates with chitinase producing ability were optimized under a set of conditions so as to conclude the possible congenial environment for chitinase production at a commercial scale.

#### 3.4.1. Colloidal Chitin Concentration

The colloidal chitin concentration considerably influenced chitinase production. Chitinase activity was observed significantly highest at 2% colloidal chitin concentration in most of the isolates except isolates AT2, AT7 and BT7, where the chitinase activity was observed to be highest at 1.5% concentration (Table 2). Initially, the chitinase activity increases with the increase in colloidal concentration and then activity falls slightly.

#### 3.4.2. Effect of pH and Temperature

Our studies recorded that pH had a statistically significant impact on the chitinase production process. Average maximum chitinase production was recorded at pH 6.0. All the isolates followed a similar trend except BT1 and BT8, where it was maximum at pH 6.5 (Table 3).

Similarly, the chitinase production increased significantly in all the isolates with an increase in temperature from 15 to 30 °C and decreased beyond this temperature. Significantly maximum average chitinase activity was observed at 30 °C. All the isolates followed a similar trend with the exception of isolate BT3, in which peak production occurred at 25 °C (Table 4).

#### 3.4.3. Effect of Nitrogen and Carbon Sources

**Figure 4:**
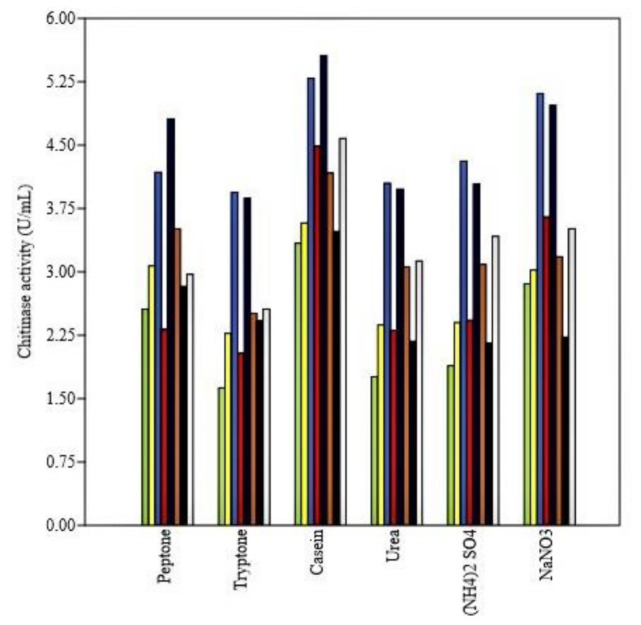
Effect of nitrogen sources on chitinase activity of various *Trichoderma* isolates.Yellow-green: AT2; Yellow: AT3; Royal blue: AT7; Red: BT1; Dark blue: BT3; Chocolate: BT4; Black: BT7 and Gainsboro: BT8.

Nitrogen sources have resulted in a statistically significant influence on enzyme production in all the isolates. The average highest production of chitinase was observed in casein (4.31 U/mL) and lowest in tryptone (2.65 U/mL). The bar chart (Figure 4) reflects the variation among isolates (different colors) in chitinase production with different nitrogen sources and highest enzyme activity reaching as significantly highest as 5.56 units/mL in isolate BT3 when casein was used as nitrogen source.

**Figure 5:**
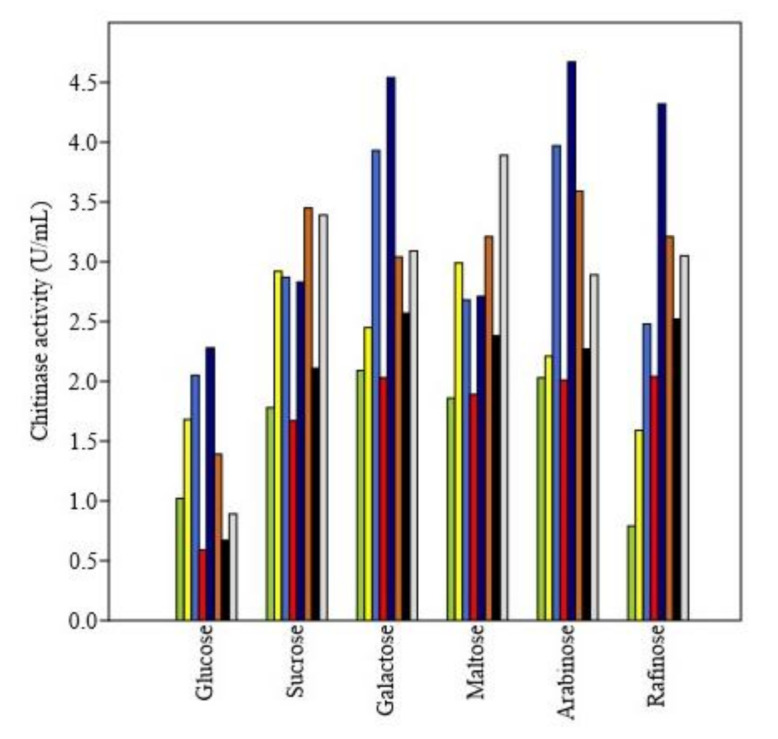
Effect of carbohydrate supplementations on chitinase activity. Yellow-green: AT2; Yellow: AT3; Royal blue: AT7; Red: BT1; Dark blue: BT3; Chocolate: BT4; Black: BT7 and Gainsboro: BT8.

The experiment on the effect of various carbohydrate sources on chitinase production revealed that all the carbohydrate sources caused a statistically significant impact on chitinase production among all the isolates. The average chitinase activity was significantly highest in galactose (2.96 U/mL) and least in glucose (1.32 U/mL) supplementations. Raffinose caused the least chitinase production in the isolates AT2 (0.79 U/mL) and AT3 (1.59 U/mL), and in all other isolates, glucose caused the least chitinase production when glucose supplementation was done (Figure 5).

#### 3.4.4. Effect of Metal Ions on Chitinase Activity of *Trichoderma* Species

While assessing the impact of various metal ion supplementations on chitinase activity, it was noted that the enzyme activities increased significantly in the presence of Na^+^, Mg^2+^ and Ca^2+^, whereas the activities were markedly inhibited by Zn^2+^, Hg^2+^, Fe^2+^, Ag^+^ and K^+^ (Table 5). The significantly average highest and lowest chitinase activity was observed upon supplementation with Mg^2+^ and Hg^2+^ ions, respectively. Among all the isolates, the significantly highest activity of chitinase among all the isolates was observed in isolate BT7 (173.44 U/mL). 

### 3.5. Biocontrol Activity of Culture Filtrates under In Vitro Conditions

**Figure 6:**
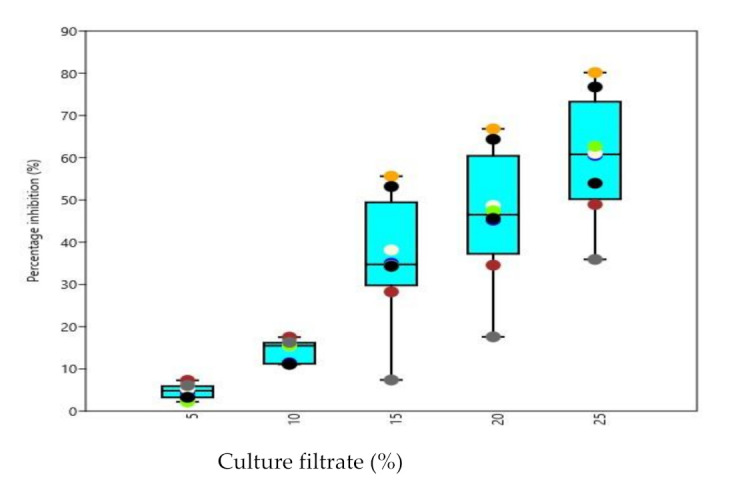
Box and Jitter plot representing Percentage inhibition of *D. necatrix* by various chitinase producing *Trichoderma* isolates. [Symbols in the plot represent: AT2: Orange; AT3: Black; AT7: Blue; BT1: Brown; BT3: Cornsilk; BT4: Chartreuse; BT7: Dim gray; BT8: Indigo].

**Figure 7:**
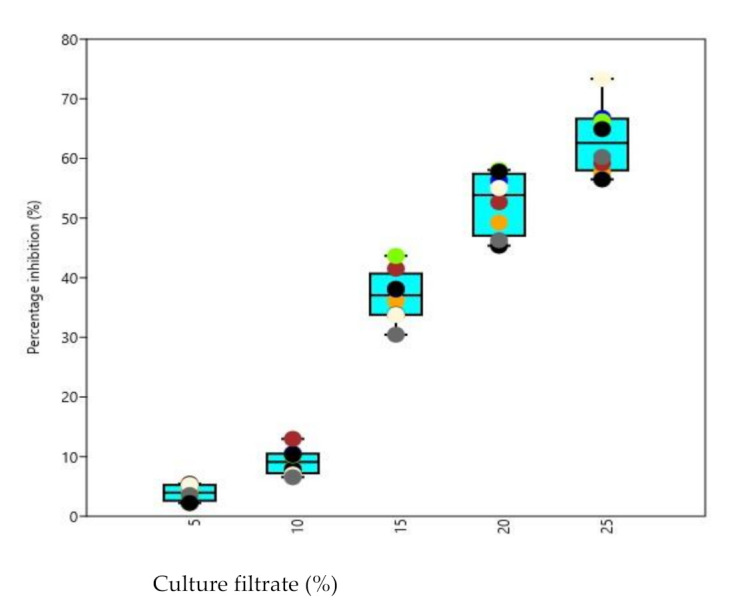
Box and Jitter plot representing percentage inhibition of *F. oxysporum* by various chitinase producing *Trichoderma* isolates. [Symbols in the plot represent: AT2: Orange; AT3: Black; AT7: Blue; BT1: Brown; BT3: Cornsilk; BT4: Chartreuse; BT7: Dim gray; BT8: Indigo].

**Figure 8:**
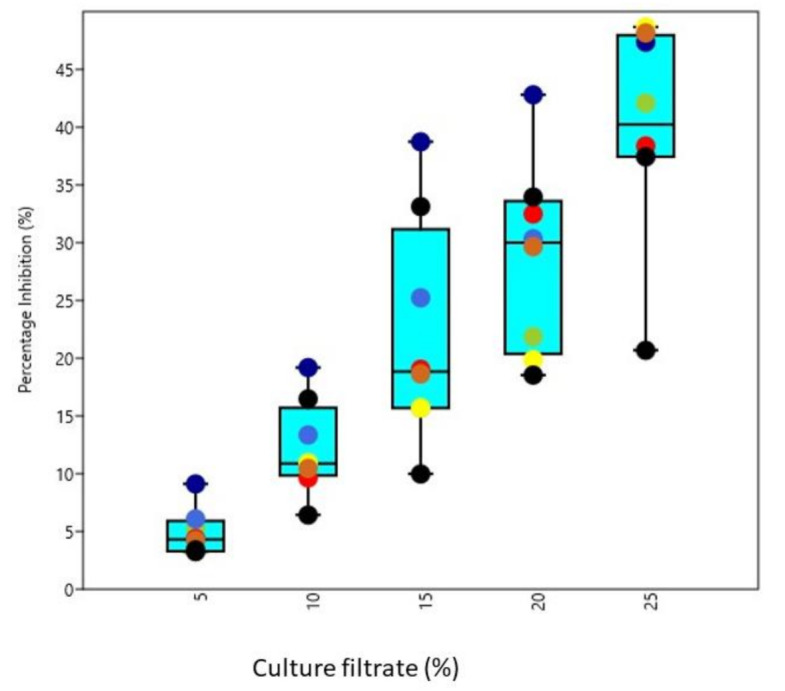
Box and Jitter plot representing percentage inhibition of *F. solani* by various chitinase producing *Trichoderma* isolates. [Symbols in the plot represent: AT2: Orange; AT3: Black; AT7: Blue; BT1: Brown; BT3: Cornsilk; BT4: Chartreuse; BT7: Dim gray; BT8: Indigo].

**Figure 9:**
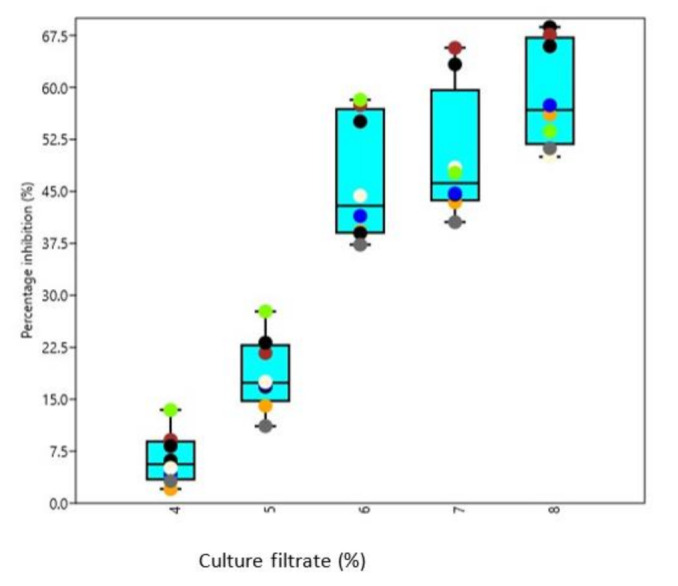
Box and Jitter plot representing percentage inhibition of *P. aphanidermatum* by various chitinase producing *Trichoderma* isolates. [Symbols in the plot represent: AT2: Orange; AT3: Black; AT7: Blue; BT1: Brown; BT3: Cornsilk; BT4: Chartreuse; BT7: Dim gray; BT8: Indigo].

All the test phytopathogens viz., *Dematophora necatrix, Fusarium oxysporum, Fusarium solani* and *Pythium aphanidermatum*were significantly inhibited by chitinase producing isolated *Trichoderma* strains, concentrations and isolates differed significantly from each other among all the tested pathogens. The observations recorded revealed that maximum inhibition of *D. necatrix* at 25% culture-free filtrate concentration was significantly caused by isolate AT2 (80.17%), while as at 5% culture-free filtrate concentration (Figure 6), the maximum inhibition was caused by BT1 (7.29%). In the same way, the maximum inhibition of *F. oxysporum* at 25% culture-free filtrate concentration (Figure 7) was caused by isolate BT3 (73.34%), while at 5% culture-free filtrate concentration, the maximum inhibition was caused by AT3 (5.44%). The maximum inhibition (Figure 8) of *F. solani* at 25% culture-free filtrate concentration was caused by isolate AT3 (48.67%), while at 5% culture-free filtrate concentration, the maximum inhibition was caused by BT3 (9.12%) and maximum inhibition of *P. aphanidermatum* at 25% culture-free filtrate concentration (Figure 9) was caused by isolate BT2 (77.38%), while at 5% culture-free filtrate concentration, the maximum inhibition was caused by BT4 (13.44%).

### 3.6. Biocontrol Activity of Culture Filtrates under In Vivo Conditions

All the isolates inhibited wilt incidence in the chilli crop in the pot culture experiment; however, the variation among all the isolates was statistically significant. The lowest wilt incidence and highest disease control were observed when using isolate BT4 with mean wilt incidence and disease control of 21% and 48.05%, respectively. Upon inoculation with isolate AT5, mean wilt incidence and disease control remained 22.62% and 44.05%, respectively. The highest wilt incidence of 40.43%, and no disease control was recorded in the treatment where no culture filtrate was applied. Among the *Trichoderma* isolates, the inoculation with isolate BT7 proved least effective, with wilt incidence of 31.39% and disease control of only 22.35% (Table 6). All the treatments were statistically significant.

## 4. Discussion

*Trichoderma* is one of the most important avirulent opportunistic symbionts associated with plants across families. Besides being a symbiont, the potential of *Trichoderma* sp. in almost all the biological management of phytopathogen strategies is well-founded. These attributes have made it a unique, cost-effective and ecologically sound tool for phytopathogen suppression. Being the native candidate to most of the soil niches, it is known for not disturbing soil microflora equilibrium. Being extremely versatile in metabolite production has made this genus the most interesting entity to researchers across the world. Chitinase mediated phytopathogen containment being the central mechanism to its mode of action, has been investigated in this research. A total of 110 *Trichoderma* isolates were collected from different kitchen gardens in J&K, India. However, we restricted our studies only to 17 isolates based on their capability to inhibit the growth of potential chilli pathogen (*F. solani*) of the area. The results on screening of chitinolytic strains revealed that the chitinase activity among all the screened isolates varied between 1.13 U/mL to 3.38 U/mL. The *Trichoderma* sp. have previously been isolated from the different soils [27]. The *Trichoderma* isolates have a founded footprint in the rhizosphere of plants and, as such, may be considered to be the most appropriate site for its isolation [28]. The investigations on chitinase production by *Trichoderma* sp. have found huge diversity among the isolates in this context, and production levels were as low as 0.061 U/mL [3], although many investigations have reported the chitinase production in the range to substantiate our findings [29,30]. In some investigations, the production levels have reached as high as 37 U/mL in some fungal biocontrol agents [31]. The results of the present study revealed that *T. harzianum* has a huge morphological diversity. This could be a result of the different environmental conditions predominant in the niches they occupy [3]. To confirm the identity of genus and species at the molecular level, the ITS region was amplified, and to further validate the ITS results, two genes, *rpb2* and *tef*1α, were used, which amplified the products of 1000bp and 800bp, respectively, from all the isolates. The *tef*1α gene results showed sequence similarity of 98% with *T. harzianum,* whereas *rpb2* showed 99% with *T. afroharzianum* (cryptic species of *T. harzianum),* respectively. Likewise, our investigation of the importance of *rpb2 and tef*1α in validating the ITS results has been realized by many investigators [28].

It has been suggested that *T. harzianum* is the collective name of a set of asexual fungal strains which exhibit heterogeneity in genome structure, DNA sequence and behavior [32]. In contrast to the very high similarity between ITS1 and ITS2 in most of the taxa, a significantly high variation is displayed by *T. harzianum* in ITS1 and ITS2 sequences [33]. [34], from his studies on diversity in *T. harzianum* based on four gene sequences concluded that *T. harzianum* is a species complex lacking morphologically distinct diagnostic characters [35].

Studies on the effect of colloidal chitin concentration on chitinase production revealed that initially, the chitinase activity increases with the increase in colloidal concentration, and then activity falls slightly. Chitinase activity was observed significantly highest at 2% colloidal chitin concentration in most of the isolates except isolates AT2, AT7 and BT7, where the chitinase activity was observed to be highest at 1.5% concentration. Our findings are supported by the findings of [36], who observed a direct correlation between colloidal chitin concentration and chitinase production up to a certain level. A decrease in the chitinase activity beyond a certain concentration of colloidal chitin may be a result of the accumulation of intermediates that result from chitin degradation into the medium leading to the buildup of a synthetic inhibitor of chitinase itself [37]. A majority of the fungus secretes chitinase at neutral or slightly acidic pH conditions [38]. Our studies recorded optimum chitinase production at pH 6.0 in a majority of the isolates, except BT1 and BT8, where it was maximum at pH 6.5. Acidic pH has been reported to be an important factor in the chitinase production of *Trichoderma* [39]. At higher pH levels, the stability of the chitinase enzyme reduces by 57 to 67% [40], conferring further a foundation to our findings. Similar findings have been reported by many other workers [41]. The nitrogen sources can also affect the pH of the medium, which in turn may influence the activity and stability of the enzyme [42].

Maximum chitinase activity was observed at 30 °C with the exception of isolate BT3, in which peak production occurred at 25 °C. The highest chitinase activity of the *Penicillium oxalicum* genus has been previously reported in the same temperature range [31]. Our results are further supported by the investigations of other authors [43,44]. Several *Trichoderma* sp. are capable of yielding voluminous enzymes like chitinase, which bring about the toxicity or cell wall degradation to many fungal phytopathogens, prevalently soil-borne. However, the production levels of these enzymes hinge on many factors such as pH, incubation periods, carbon sources, temperature, and nitrogen sources [45].

In the present investigation, nitrogen sources have led to a significant influence on enzyme production in all the isolates. The average highest production of chitinase was observed in casein and lowest in tryptone. The inorganic nitrogen sources proved less favorable for chitinase production. This is expected as organic nitrogen sources (casein) are rich in amino acids and short peptides that support the enzyme production process. Casein yielded the highest enzyme activity in all the isolates of *Trichoderma*. Casein contains some essential amino acids as well as some carbohydrates and some inorganic elements like calcium and phosphorus. The investigation on optimization of chitinase production by *Trichoderma* sp. by [45] observed the higher chitinase activity upon organic nitrogen amendments to the production media, thus supporting our findings in this context.

The experiment on the effect of various carbohydrate sources on chitinase production revealed that all the carbohydrate sources caused a significant impact on chitinase production among all the isolates. The average chitinase activity was significantly highest in galactose and least in glucose supplementations. Raffinose caused the least chitinase production in some of the isolates. The decrease in chitinase activity upon carbon source supplementations indicates that there could be repression of enzyme production by these carbon sources. The expression of chitinase synthesizing genes has been reported to be downregulated by the presence of an extra carbon source like glucose through catabolite repression [46]. Our findings are also supported by [45].

While assessing the impact of various metal ion supplementations on chitinase activity, it was noted that the enzyme activities increased significantly in the presence of Na^+^, Mg^2+^ and Ca^2+^, whereas the activities were markedly inhibited by Zn^2+^, Hg^2+^, Fe^2+^, Ag^+^ and K^+^. The significantly average highest and lowest chitinase activity was observed upon supplementation with Mg^2+^ and Hg^2+^ ions, respectively. Similar results in context to Fe^2+^, Ag and contrary in context to Na^+^ were reported by [31] in the *Trichoderma* genus. These findings could be attributed to a change in electrostatic bonding that changed the tertiary structure of enzymes and hence their activity [47]. The inhibition of chitinases by Zn^2+^ and Hg^2+^ could be related to the residues of aspartic and glutamic acid in chitinases [31].

Studies on the effect of culture filtrate on the growth of some prominent phytopathogens *viz., D. necatrix, F. oxysporum, F. solani* and *P. aphanidermatum* revealed that their growth was significantly inhibited by chitinase producing isolated *Trichoderma* strains, concentrations and isolates differed significantly from each other among all the tested pathogens. Growth inhibition of the pathogens by *Trichoderma* culture-free filtrate has been reported by several workers [20,28]. The effect of culture filtrate of *Trichoderma* on the pathogen might be due to the production of chitinase [3,48] found that culture filtrates of *T. harzianum* inhibit zoospore germination, germ tube elongation and mycelial growth of *P. aphanidermatum,* causing damping-off disease of tobacco. The extracellular enzymes produced by *Trichoderma* strains may be correlated with the antagonism. *Trichoderma* directly attacks the plant pathogen by producing lytic enzymes such as chitinases [12]. These features of T. *harzianum* could be attributed to the reduced wilt incidence and highest disease control observed under hothouse conditions in the present investigation.

## 5. Conclusions

The present study confirms the presence of chitinase producing strains of *Trichoderma* in temperate vegetable growing areas of India. It also confirms that the chitinase producing ability of *Trichoderma* strains can be increased by different types of manipulations in cultivation practices. *Trichoderma* species have been proven to perform effectively in managing various soil-borne pathogens affecting various field crops under temperate conditions. Following the isolation and identification of *Trichoderma* species, we found that *T. harzianum* and *T. afroharzianum* were the dominant species in the rhizosphere of vegetable crops in Kashmir, India. Their excellent biocontrol results mean that *T. afroharzianum* strains BT4 and AT5 could be used as the preferred *Trichoderma* strains for the biocontrol of wilt disease of chilli and other vegetable crops. This study provided scientific guidance for the biocontrol of chilli wilt disease, which is threatening vegetable cultivation in Kashmir. The future endeavors of research on the aspect shall be to purify and characterize the diverse possible chitinase types among the isolates and identifying the bottlenecks in their production process at the molecular level. The isolated strains need to be tested on many other crops after extensive standardization of their production process at the commercial scale.

## Figures and Tables

**Table 1:** Chitinase activity of various *Trichoderma* isolates.

S. No.	Isolate	Conidiophore Branching	Conidial Color	Species Identified	Chitinase Activity (Units/mL ± SD)
1.	AT1	Highly branched regular	Green	*Trichoderma harzianum*	ND *
2.	AT2	Highly branched regular	Green	*T. harzianum*	1.13 ± 0.04 ^g^
3.	AT3	Highly branched regular	Green	*T. harzianum*	2.06 ± 0.10 ^d^
4.	AT4	Highly branched regular	Green	*T. harzianum*	ND
5.	AT5	Highly branched regular	Green	*T. harzianum*	ND
6.	AT6	Highly branched regular	Green	*T. harzianum*	ND
7.	AT7	Highly branched regular	Green	*T. harzianum*	3.26 ± 0.13 ^b^
8.	BT1	Highly branched regular	Green	*T. harzianum*	1.19 ± 0.08 ^g^
9.	BT2	Highly branched regular	Green	*T. harzianum*	ND
10.	BT3	Highly branched regular	Green	*T. harzianum*	3.38 ± 0.22 ^a^
11.	BT4	Highly branched regular	Green	*T. harzianum*	2.07 ± 0.13 ^c^
12.	BT5	Highly branched regular	Green	*T. harzianum*	ND
13.	BT6	Highly branched regular	Green	*T. harzianum*	ND
14.	BT7	Highly branched regular	Green	*T. harzianum*	1.71 ± 0.14 ^f^
15.	BT8	Highly branched regular	Green	*T. harzianum*	2.04 ± 0.08 ^e^
16.	BT9	Highly branched regular	Green	*T. harzianum*	ND
17.	BT10	Highly branched regular	Green	*T. harzianum*	ND

Means in the column with the same letters didn’t differ by Tukey’s test. * ND = Not Determined.

**Table 2:** Effect of colloidal chitin concentration on chitinase activity of *Trichoderma* isolates.

Isolates	Chitin Concentration (%)				
	0.5	1.0	1.5	2.0	2.5
AT2	1.33 ± 0.12 ^h^	1.47 ± 0.04 ^h1^	2.53 ± 0.11 ^f2^	2.10 ± 0.06 ^h3^	1.93 ± 0.03 ^h3^
AT3	2.11 ± 0.08 ^e^	2.48 ± 0.14 ^g1^	2.94 ± 0.04 ^d2^	3.03 ± 0.03 ^e3^	2.38 ± 0.04 ^h4^
AT7	3.51 ± 0.13 ^a^	3.67 ± 0.10 ^b1^	3.89 ± 0.09 ^a2^	3.11 ± 0.09 ^d3^	2.74 ± 0.04 ^d4^
BT1	1.57 ± 0.18 ^g^	2.05 ± 0.04 ^f1^	2.33 ± 0.03 ^h2^	2.89 ± 0.05 ^f3^	2.31 ± 0.04 ^f4^
BT3	3.46 ± 0.14 ^b^	3.69 ± 0.14 ^a1^	3.82 ± 0.03 ^b2^	4.32 ± 0.06 ^a3^	3.75 ± 0.02 ^b4^
BT4	2.42 ± 0.14 ^d^	2.65 ± 0.29 ^d1^	2.91 ± 0.06 ^e2^	3.48 ± 0.6 ^c3^	2.79 ± 0.08 ^c4^
BT7	1.93 ± 0.05 ^f^	2.05 ± 0.04 ^e1^	2.45 ± 0.09 ^g2^	2.12 ± 0.03 ^g3^	2.03 ± 0.05 ^g4^
BT8	2.84 ± 0.11 ^c^	3.42 ± 0.2 ^c1^	3.69 ± 0.04 ^c2^	4.21 ± 0.06 ^b3^	3.91 ± 0.06 ^a4^

Means in the column with same letters didn’t differ by Tukey’s test.

**Table 3:** Effect of pH on chitinase activity of various *Trichoderma* isolates.

Isolates	(a)								
	3.0	3.5	4.0	4.5	5.0	5.5	6.0	6.5	7.0
**AT2**	0.57 ± 0.12 ^f^	0.73 ± 0.06 ^g1^	0.99 ± 0.03^h2^	1.23 ± 1.23^h3^	1.72 ± 0.05 ^g4^	1.93 ± 0.04^ah5^	2.04 ± 0.05 ^h6^	1.94 ± 0.06 ^h7^	1.82 ± 0.04 ^g8^
**AT3**	1.07 ± 0.06 ^e^	1.37 ± 0.08 ^e1^	1.84 ± 0.02 ^e2^	1.97 ± 1.97 ^e3^	2.37 ± 0.06 ^f4^	2.79 ± 0.05 ^f5^	3.07 ± 0.03^f6^	2.81 ± 0.05 ^f7^	2.38 ± 0.03 ^f8^
**AT7**	2.15 ± 0.61^b^	2.69 ± 0.04 ^b1^	2.95 ± 0.03^b2^	3.28 ± 3.28 ^a3^	3.67 ± 0.05^a4^	4.01 ± 0.03 ^a5^	5.18 ± 0.05^a6^	4.11 ± 0.06 ^b7^	4.11 ± 0.02 ^a8^
**BT1**	0.32 ± 0.05 ^h^	0.71 ± 0.02 ^h1^	1.03 ± 0.04 ^g2^	1.41 ± 1.41^g3^	1.73 ± 0.09 ^h4^	1.98 ± 0.03 ^ag5^	2.31 ± 0.04^g6^	2.43 ± 0.04 ^g7^	2.14 ± 0.03 ^e8^
**BT3**	2.28 ± 0.51^a^	2.81 ± 0.07 ^a1^	3.05 ± 0.02^a2^	3.22 ± 3.22 ^b3^	3.59 ± 0.06 ^b4^	3.97 ± 0.04 ^b5^	4.67 ± 0.05 ^b6^	4.05 ± 0.05 ^c7^	3.62 ± 0.05^b8^
**BT4**	1.29 ± 0.09 ^d^	1.43 ± 0.09 ^d1^	1.93 ± 0.04 ^d2^	2.12 ± 2.12^d3^	2.39 ± 0.04 ^e4^	2.83 ± 0.05 ^e5^	3.35 ± 0.05 ^d6^	2.93 ± 0.04 ^e7^	2.67 ± 0.05 ^d8^
**BT7**	0.45 ± 0.09 ^g^	0.87 ± 0.16 ^f1^	1.32 ± 0.04 ^f2^	1.89 ± 1.89 ^f3^	2.41 ± 0.04 ^d4^	2.91 ± 0.05 ^d5^	3.21 ± 0.05 ^e6^	3.04 ± 0.03 ^d7^	2.93 ± 0.05 ^d8^
**BT8**	2.11 ± 0.61 ^c^	2.46 ± 0.04^c1^	2.67 ± 0.07 ^c2^	2.82 ± 2.82^c3^	3.05 ± 0.06^c4^	3.47 ± 0.04 ^c5^	3.98 ± 0.03^c6^	4.12 ± 0.05 ^a7^	3.37 ± 0.06 ^c8^

Means in the column with same letters didn’t differ by Tukey’s test.

**Table 4:** Effect of temperature (°C) on chitinase activity of various *Trichoderma* isolates.

Isolates	15	20	25	30	35	40	45
AT2	0.37 ± 0.13^h^	0.93 ± 0.10 ^g1^	1.46 ± 0.13 ^g2^	1.59 ± 0.28 ^g3^	0.88 ± 0.12 ^h4^	0.62 ± 0.05 ^h5^	0.39 ± 0.06^h6^
AT3	1.29 ± 0.29 ^b^	1.69 ± 0.29 ^d1^	2.28 ± 0.24 ^d2^	2.89 ± 0.06 ^c3^	2.39 ± 0.12 ^b4^	2.11 ± 0.10 ^b5^	1.95 ± 0.04 ^b6^
AT7	2.59 ± 0.28 ^a^	2.94 ± 0.08 ^a1^	3.37 ± 0.26 ^b2^	3.59 ± 0.28 ^a3^	3.01 ± 0.01 ^a4^	2.43 ± 0.09 ^a5^	2.04 ± 0.04 ^a6^
BT1	0.67 ± 0.11^g^	0.92 ± 0.09 ^h1^	1.27 ± 0.13 ^h2^	2.22 ± 0.19 ^f3^	1.66 ± 0.28 ^g4^	1.23 ± 0.11 ^g5^	0.94 ± 0.10 ^e6^
BT3	2.22 ± 0.18 ^c^	2.91 ± 0.25 ^b1^	3.46 ± 0.17 ^a2^	2.89 ± 0.06 ^c3^	1.67 ± 0.29 ^f4^	1.47 ± 0.13 ^e5^	1.38 ± 0.09 ^c6^
BT4	1.06 ± 0.08^f^	1.16 ± 0.14 ^f1^	2.22 ± 0.18 ^e2^	2.87 ± 0.06^d3^	2.16 ± 0.11 ^c4^	1.69 ± 0.29 ^c5^	1.51 ± 0.20 ^d6^
BT7	1.47 ± 0.21 ^d^	1.72 ± 0.20 ^c1^	1.89 ± 0.13 ^f2^	2.42 ± 0.04 ^e3^	1.82 ± 0.10 ^e4^	1.26 ± 0.12^f5^	0.59 ± 0.06 ^f6^
BT8	1.35 ± 0.21 ^e^	1.51 ± 0.50 ^e1^	2.44 ± 0.04 ^c2^	2.97 ± 0.01 ^b3^	1.96 ± 0.10 ^d4^	1.35 ± 0.32 ^d5^	0.55 ± 0.05 ^g6^

Means in the column with the same letters didn’t differ by Tukey’s test.

**Table 5:** Effect of metal ions and inhibitors on chitinase activity of *Trichoderma* isolates.

Isolates	Relative Activity (%) ± SD								
	Control	KCl	NaCl	AgNO_3_	CaCl_2_	FeSO_4_	HgCl_2_	MgCl_2_	Zn SO_4_
AT2	100	97.32 ± 1.59 ^d^	98.31 ± 2.05 ^a1^	78.07 ± 3.01 ^e2^	128.67 ± 2.15 ^g3^	84.48 ± 0.72 ^c4^	71.21 ± 1.71 ^a5^	135.99 ± 3.85 ^g6^	93.53 ± 1.47 ^a7^
AT3	100	89.05 ± 0.06 ^h^	105.23 ± 1.21^a1^	76.69 ± 3.28^f2^	156.23 ± 1.33 ^c3^	79.28 ± 1.03 ^e4^	52.02 ± 0.92 ^b5^	161.33 ± 2.71^c6^	63.36 ± 3.17 ^b8^
AT7	100	98.02 ± 1.61^c^	98.89 ± 1.96 ^a1^	81.72 ± 2.84^c2^	134.67 ± 1.00 ^e3^	81.41 ± 1.39 ^d4^	37.79 ± 0.91 ^f5^	147.72 ± 2.10 ^d6^	46.35 ± 2.06 ^f8^
BT1	100	98.89 ± 1.96^a^	104.61 ± 1.21 ^a1^	75.45 ± 3.46^h2^	156.32 ± 1.54 ^b3^	76.92 ± 1.00 ^g4^	50.29 ± 2.41 ^c5^	162.33 ± 2.35 ^b6^	59.63 ± 3.18 ^d8^
BT3	100	93.54 ± 1.31 ^f^	102.20 ± 1.20 ^a1^	80.58 ± 2.07^d2^	134.35 ± 2.51 ^f3^	84.72 ± 0.74 ^b4^	21.47 ± 3.12 ^h5^	137.12 ± 2.51 ^f6^	31.45 ± 4.16 ^i8^
BT4	100	97.10 ± 0.65 ^e^	98.83 ± 1.97 ^a1^	84.38 ± 1.53^b2^	117.22 ± 1.33 ^g3^	89.33 ± 1.13 ^a4^	49.39 ± 2.06 ^d5^	125.31 ± 2.69 ^h6^	61.73 ± 2.54 ^c8^
BT7	100	98.38 ± 2.05^b^	101.25 ± 1.00 ^a1^	85.08 ± 1.00^a2^	159.59 ± 2.04^a3^	77.18 ± 1.82 ^f4^	25.61 ± 2.08 ^g5^	173.44 ± 3.49^a6^	42.04 ± 2.73 ^g8^
BT8	100	92.34 ± 1.46 ^g^	107.05 ± 0.23 ^a1^	75.56 ± 0.83^g2^	152.29 ± 1.71 ^d3^	71.61 ± 1.48 ^h4^	39.77 ± 0.87 ^e5^	136.18 ± 3.46 ^e6^	51.78 ± 3.32 ^e8^

Means in the column with the same letters didn’t differ by Tukey’s test.

**Table 6:** Effect of culture-free filtrates of *Trichoderma* isolates on wilt incidence of chilli cv. Kashmir long 1 in pot culture.

Isolates	Wilt Incidence (%)				Disease Control (%)
	Flowering	Fruit Formation	Ripening	Mean	
AT1	4.20 ± 0.21 ^q^	24.35 ± 1.34 ^n1^	44.26 ± 1.85 ^p2^	24.27 ^p3^	39.97 ^c4^
AT2	5.80 ± 0.52 ^g^	26.32 ± 1.15 ^h1^	50.32 ± 1.26 ^h2^	27.48 ^i3^	32.03 ^k4^
AT3	6.80 ± 0.36 ^c^	27.31 ± 1.35 ^g1^	52.16 ± 0.89 ^d2^	28.73 ^c4^	28.86 ^q4^
AT4	5.20 ± 0.14 ^m^	25.68 ± 0.88 ^k1^	47.56 ± 1.02 ^m2^	26.15 ^k3^	35.32 ^h4^
AT5	5.30 ± 0.21 ^l^	22.13 ± 1.63 ^p1^	40.45 ± 1.08 ^q2^	22.62 ^q3^	44.05 ^b4^
AT6	4.60 ± 0.28 ^o^	25.00 ± 0.87 ^l1^	47.80 ± 0.71 ^l2^	25.80 ^m3^	36.18 ^f4^
AT7	4.80 ± 0.46 ^n^	25.54 ± 1.22 ^l1^	49.14 ± 0.94 ^k2^	26.49 ^j3^	34.47 ^j4^
BT1	5.30 ± 0.29 ^k^	26.10 ± 1.3 ^i1^	51.21 ± 0.92 ^g2^	27.54 ^h3^	31.88 ^l4^
BT2	4.10 ± 0.14 ^r^	24.47 ± 1.75 ^m1^	46.62 ± 1.26 ^n2^	25.06 ^n3^	38.01 ^d4^
BT3	6.10 ± 0.38 ^e^	26.51 ± 1.25 ^e1^	52.10 ± 0.76 ^e2^	28.24 ^f3^	30.15 ^n4^
BT4	3.50 ± 0.57 ^j^	19.65 ± 1.22 ^r1^	39.84 ± 0.62 ^r2^	21.00 ^r3^	48.05 ^a4^
BT5	5.70 ± 0.36 ^h^	25.84 ± 1.50 ^j1^	53.45 ± 1.31 ^c2^	28.33 ^e3^	29.92 ^o4^
BT6	4.25 ± 0.37 ^p^	23.41 ± 1.40 ^o1^	50.12 ± 1.16 ^i2^	25.92 ^l3^	35.88 ^i4^
BT7	6.30 ± 0.37 ^d^	29.31 ± 0.79 ^c1^	58.57 ± 0.6 ^b2^	31.39 ^b3^	22.35 ^r4^
BT8	6.00 ± 0.38 ^f^	27.46 ± 1.30 ^d1^	51.60 ± 1.21 ^f2^	28.35 ^d3^	29.87 ^p4^
BT9	5.51 ± 0.63 ^i^	21.23 ± 1.07 ^q1^	49.60 ± 1.24 ^j2^	25.44 ^o3^	37.07 ^e4^
BT10	7.41 ± 0.33 ^b^	31.35 ± 1.67 ^b1^	44.52 ± 0.77 ^o2^	27.76 ^g3^	31.33 ^m4^
Control	9.84 ± 0.89^a^	41.25 ± 1.14 ^a1^	70.20 ± 1.31 ^a2^	40.43 ^a3^	0.00

Means in the column with the same letters didn’t differ by Tukey’s test.

## Data Availability

All data generated or analyzed in this study are available within the manuscript and are available from the corresponding authors upon request.
